# Proteomics revealed novel functions and drought tolerance of *Arabidopsis thaliana* protein kinase ATG1

**DOI:** 10.1186/s12915-025-02149-3

**Published:** 2025-02-21

**Authors:** Shan Cheng, Siqi Fan, Chao Yang, Weiming Hu, Fen Liu

**Affiliations:** 1 Lushan Botanical Garden, Jiangxi Province and Chinese Academy of Sciences, Jiujiang, Jiangxi 332900 China; 2 College of Life Science, Nanchang University, Nanchang, Jiangxi 330031 China

**Keywords:** Autophagy, ATG1, Proteomic, Nitrogen deprivation, Plant stress

## Abstract

ATG1 stimulates autophagy biogenesis and serves as a gatekeeper for classical autophagy. To obtain insight into the control of autophagy by ATG1 and determine whether ATG1 has broader processes, we performed a thorough proteomics analysis on the Col-0 wild-type and *atg1abct* mutant in *Arabidopsis thaliana*. Proteomic data analysis pointed out that ATG1 has an unidentified function within the inositol trisphosphate and fatty acid metabolism. We also discovered ATG1-dependent autophagy has an emerging connection with ER homeostasis and ABA biosynthesis. Moreover, Gene Ontology terms for abiotic and biotic stress were strongly enriched in differentially abundant proteins, consistent with the reported role of canonical autophagy in these processes. Additional physiological and biochemical analysis revealed that *atg1abct* exhibited stronger drought resistance under both PEG-simulated drought treatment and natural drought stress. Results from DAB staining also indicated that *atg1abct* accumulation fewer ROS than Col-0 following drought treatment. As a result, these results illuminate previously unknown functions for ATG1 and offers novel perspectives into the underlying processes of autophagy function.

**Supplementary Information:**

The online version contains supplementary material available at 10.1186/s12915-025-02149-3.

## Introduction

Autophagy is a membrane-mediated and vacuole/lysosome-dependent biological process for material degradation and turnover that occurs in all eukaryotes [1]. In plants, autophagy is present throughout the life stages, including germination, growth, reproduction, and senescence, until death [2–4]. Normally, autophagy is at a low constitutive level in plant cells to maintain cytosolic homeostasis. Under various stress conditions, autophagy is stimulated and provides temporal burden relief [5]. In addition to degrading most intracellular materials, autophagy can also selectively degrade specific substrates [6].

Plant autophagy initiated with the formation of phagophore. The phagophore is a cup-shaped double-membrane structure that forms around the cargo, then elongates until it is surrounded by the cargo, and finally seals to generate a bilayer double-membrane vesicle called the autophagosome [7]. The autophagy process is facilitated by conserved functional groups of autophagy-related (ATG) proteins: (a) the ATG1/ATG13 complex, which assemble into active complexes to initiates autophagy [8]; (b) the phosphatidylinositol 3-kinase (PI3K) complex facilitates vesicle nucleation by generating phosphatidylinositol-3-phosphate (PI3P) and localization to the autophagosome [9]; (c) ATG2-ATG9-ATG18 complex localizes to the autophagosome precursors and promotes phagophore membrane extension, the transmembrane protein ATG9 provides membrane source for autophagosome [10, 11]; (d) two ubiquitination-like conjugation pathways (ATG5-ATG12 and ATG8-phosphatidylethanolamine (PE)) mediate phagophore closure and maturation [12]. Once complete, the mature autophagosome eventually fuses with vacuole, and releases the cargo into the vacuole, and cargo is degraded by hydrolases into small molecules for cellular reuse.

In *Arabidopsis thaliana*, the ATG1/ATG13 complex consists of the serine/threonine protein kinase ATG1 and its accessory proteins ATG11, ATG13, and ATG101, which are the most upstream regulators of autophagy. *Arabidopsis thaliana* possessed four *ATG1* gene loci (*ATG1a*, *ATG1b*, *ATG1c*, and *ATG1t*) and two *ATG13* gene loci (*ATG13a* and *ATG13b*). When nutrients were present in sufficient amounts, target of rapamycin (TOR) phosphorylated ATG13, which subsequently separated from ATG1 [13, 14]. Meanwhile, tumor necrosis factor receptor-associated factor (TRAF) family proteins TRAF1a and TRAF1b promote the RING finger E3 ligases SEVEN IN ABSENTIA OF *ARABIDOPSIS* THALIANA1 (SINAT1)/SINAT2 and SINAT6 to ubiquitinate and degrade ATG1 and ATG6 via the 26S proteasome, thereby preserving autophagic flux at a suitable cellular level [15, 16]. Sucrose non-fermenting-1 (SNF1)-related protein kinase 1 (SnRK1) inhibits TOR activity in response to stressors like nutrient deprivation, and ATG13 dephosphorylates and forms a tight complex with ATG1 and ATG101 to initiate autophagy [13, 14]. In addition to being activated to initiate autophagy, ATG1 can also regulate the autophagy process by interacting with other ATG proteins. For example, in mammals, unc-51 like kinase-1 (ULK1, the mammalian ortholog of the plant ATG1) interacts with BECN1/beclin1 (ATG6 in plants) and ATG14 to facilitate the development of autophagosomes [17, 18]. An essential target substrate of ULK1 is ATG16L1, which is responsible for microtubule-associated protein 1 light chain 3 isoforms (LC3B) (the mammalian ortholog of the plant ATG8s) clearance and drives autophagosome formation [19]. In yeast, ATG1 interacts with ATG9 which regulates phagosome membrane expansion [20]. ATG1 inhibits ATG4, which regulates the maturation of autophagosomes [21]. ATG1 may be involved in the Ykt6-mediated autophagosome-vacuole fusion process [22]. Furthermore, ATG1 interacts with ATG11 in yeast to regulate selective autophagy as well [23]. Apart from the ATG1/ULK1 targets that have been found, omics has also reported some putative ATG1/ULK1 substrates in yeast, *Beauveria bassiana*, and mammalian cells [24–26]. However, little is known about the protein profile affected by ATG1 in plants. Thus, further investigation of ATG1 affected proteins is necessary to gain a deeper comprehension of the regulation of plant autophagy process.

In this study, liquid chromatography-tandem mass spectrometry (LC–MS/MS)-based proteomics pointed out that ATG1 has an unidentified function within the inositol trisphosphate and fatty acid metabolism in *Arabidopsis thaliana*. To test the effect of ATG1 depletion on autophagy, we performed nitrogen deprivation (ND) treatment, which is considered to be the canonical and widely used method to induce autophagy in plants [27]. The results revealed that ATG1-dependent autophagy has an emerging connection with endoplasmic reticulum (ER) homeostasis and ABA biosynthesis. During in-depth analysis of the omics data, it was observed that Gene Ontology (GO) terms for abiotic and biotic stress were strongly enriched in differentially abundant proteins. Furthermore, we preliminarily demonstrated the drought tolerance of *atg1abct* by drought-related experiments. These results provide new insights into the mechanism and function caused of ATG1 in *Arabidopsis thaliana*.

## Results

### The overall composition of the proteome was impacted by nitrogen deprivation and the absence of ATG1

**Fig. 1:**
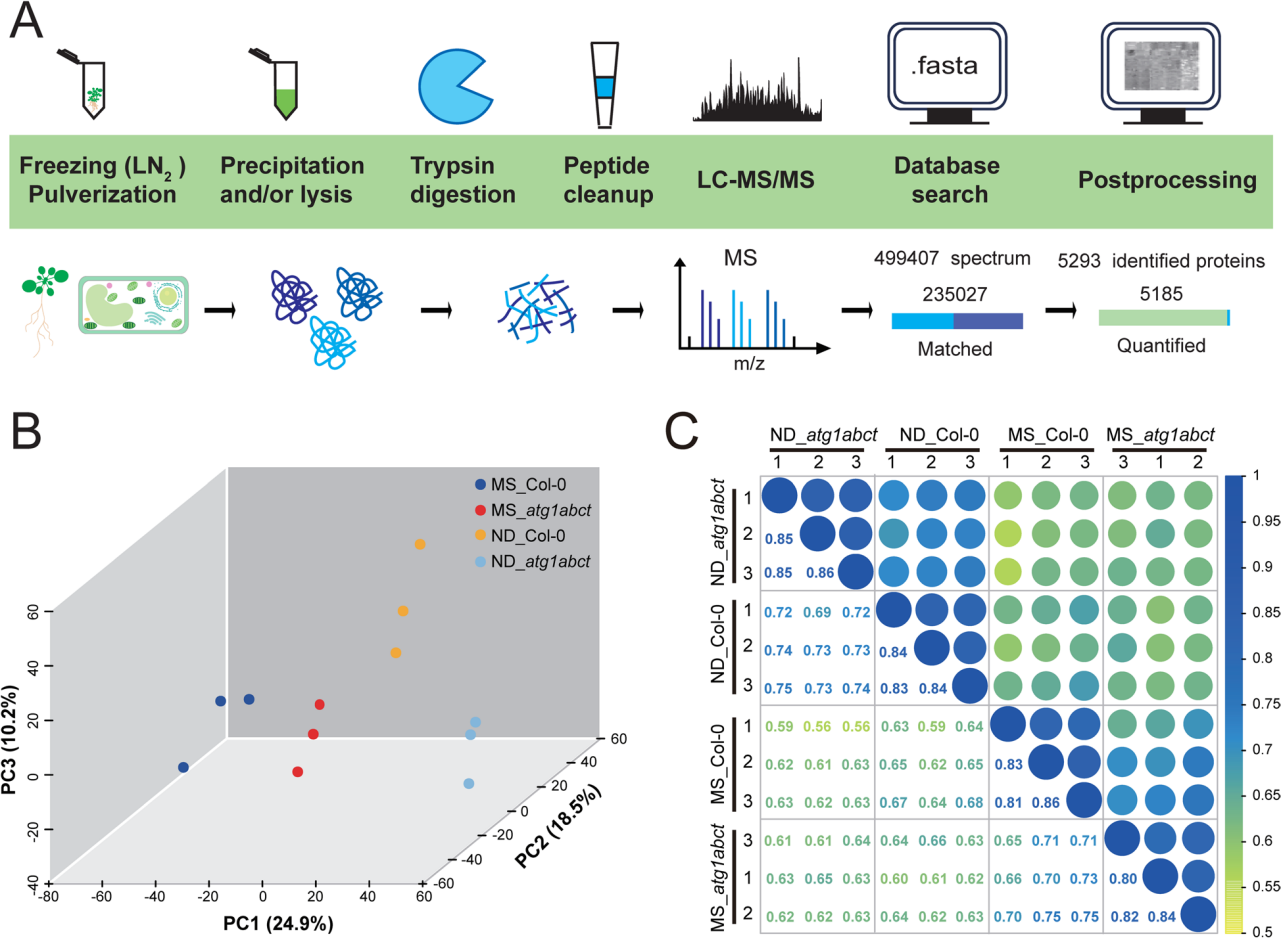
The absence of *atg1abct* and ND both contribute to differences in protein abundance in *Arabidopsis thaliana*. **A** Workflow of the proteomics analysis in this study. **B** Samples from Col-0 and *atg1abct* after ND and control treatment, respectively, formed four spatially separable clustering patterns. Based on unweighted Unifrac metrics, samples were determined using principal-component analysis (PCA). Groups are depicted with different color. **C** Sample correlation analysis was performed for all differential proteins of the samples. The selection criterion for differentially expressed proteins (DEPs) was l log_2_ fold change l > 1 and *p* values < 0.05

To thoroughly investigate the underlying mechanisms of ATG1-dependent autophagy function in plants, 4D-Label-free quantitative proteomic analysis was carried out on wild-type (Col-0) and *atg1abct* mutant [14] after 2 days of ND treatment (Fig. 1A). The proteome analysis provided the identification and quantitation of 5283 and 5185 proteins, respectively. Upon conducting quality filtering on the acquired data, principal component analysis (PCA) analysis discovered that four groups exhibited a noteworthy degree of distance (Fig. 1B). On the first component axis, nitrogen deprivation did impact plant protein expression, as evidenced by the 24.9% difference that separated the ND and control treatments (Fig. 1B). Simultaneously, sample correlation analysis was carried out for each of the four groups’ differentially expressed proteins (DEPs) (l log_2_ fold change l > 1 and *p* < 0.05), and it was determined that ND and the lack of *atg1abct* could contribute to variations in protein abundance between groups (Fig. 1C).

**Fig. 2:**
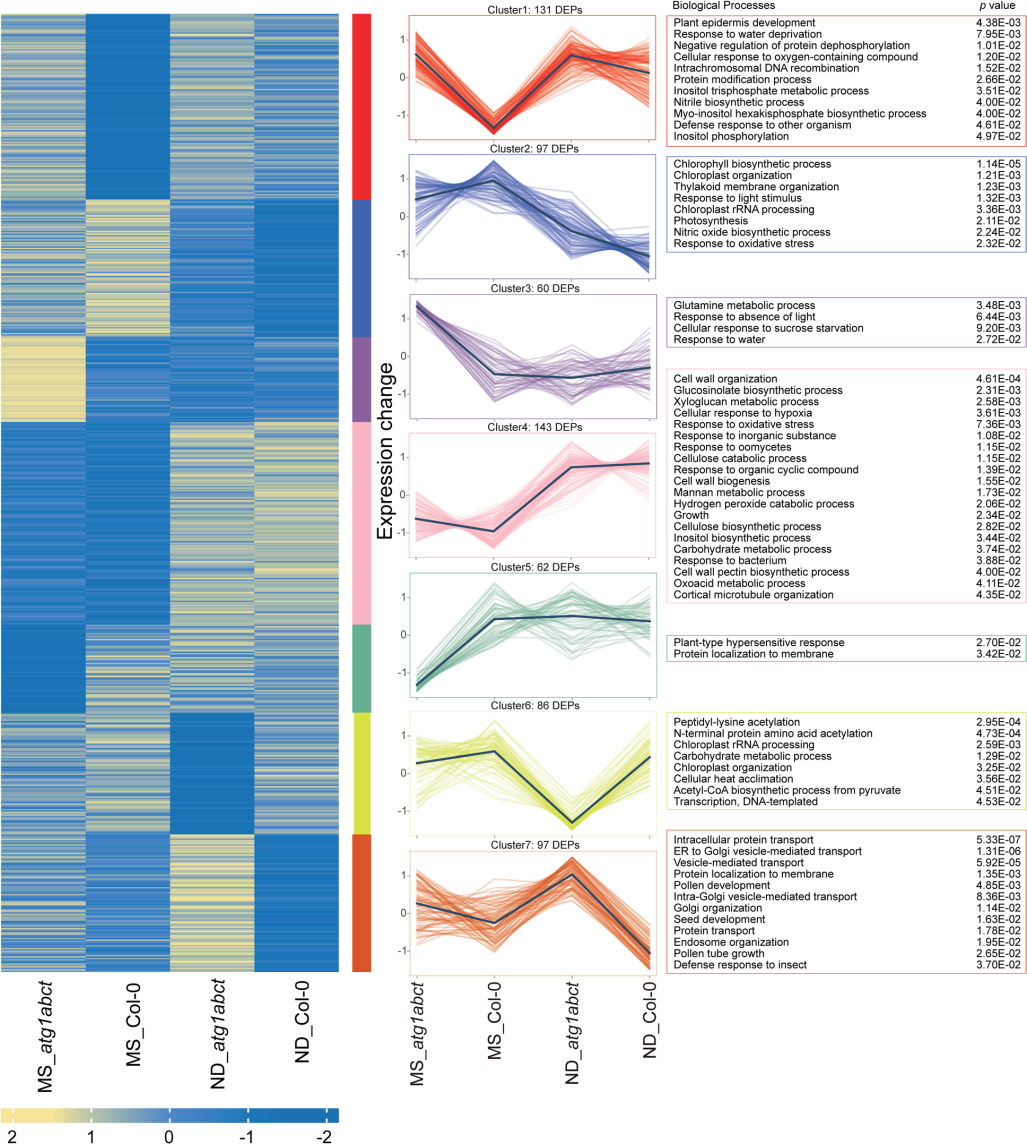
GO terms of biotic and abiotic stress response were enriched during the absence of *atg1abct* and/or ND. K-means clustering analysis of DEPs in MS_Col-0, MS_*atg1abct*, ND_Col-0, and ND_*atg1abct* groups was conducted based on their change tendencies. Each fold line of clusters 1 (*n* = 131), cluster 2 (*n* = 97), cluster 3 (*n* = 60), cluster 4 (*n* = 143), cluster 5 (*n* = 62), cluster 6 (*n* = 86), and cluster 7 (*n* = 97) indicated a change tendency of one protein. Gene Ontology (GO) database was used for functional categorization of DEPs

Using DAVID database, Gene Ontology (GO) enrichment analysis was carried out for each of the seven clusters that were created by K-means clustering analysis from all DEPs (Fig. 2 and Table S1). Of these cluster, cluster 2 showed the abundance of protein expression that was suppressed during ND treatment in both Col-0 and *atg1abct*. These proteins were mainly implicated in chloroplast and thylakoid membrane organization, photosynthesis, and nitric oxide biosynthesis, according to GO analysis. Under ND treatment, clusters 4 show the expression abundance of proteins upregulated in both Col-0 and *atg1abct*, and these proteins are mainly associated with cell wall organization and stress response. The result of GO analysis supported previous research on plant autophagy function [28–30]. Namely, GO terms with an association to stress responses and immunity were enriched (Fig. 2), such as response to water deprivation (GO:0009414), response to oxidative stress (GO:0006979), and response to bacterium (GO:0009617). This suggests that ND alters how plant react to both abiotic and biotic stresses. It was observed that the abundance of proteins in cluster 1 and 7 was upregulated in *atg1abct* mutants under ND and control treatments. These proteins were linked to response to water deprivation, defense response to insect, Inositol trisphosphate metabolism, protein modification, and transport. This demonstrates that stress response, phosphatidylinositol metabolism, protein modification, and transport may be affected by ATG1. Using CLELLO to predict subcellular localization of DEPs, most of them were found in the cytoplasm and nucleus (Fig. S1). Furthermore, when compared to the other three comparison groups, the proportion of nuclear of MS_*atg1abct*/MS_Col-0 increased while the proportion of plasma membrane decreased. These results indicate that ND and the lack of *atg1abct* cause significant changes in the plant proteomic profiles.

### The absence of ATG1 impacted plant metabolism and stress response related proteins

**Fig. 3:**
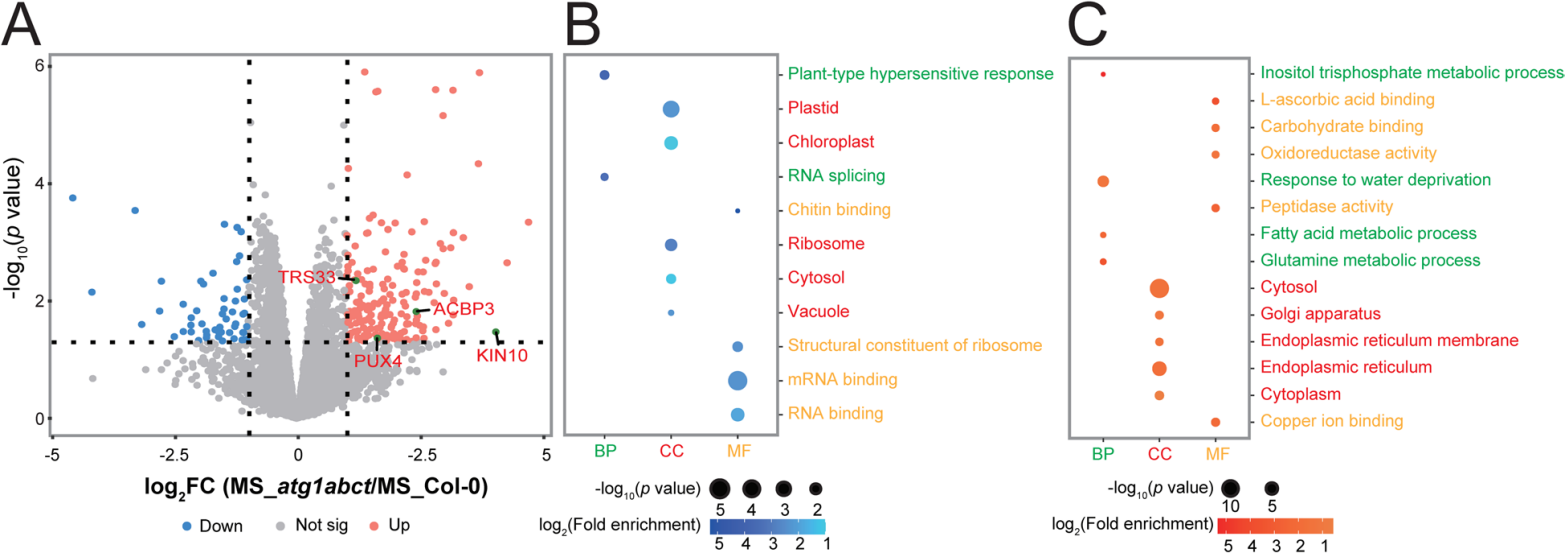
*atg1abct* mutant mainly affects plant metabolism and stress response related proteins. **A** Volcano plots show the preferential accumulation of individual proteins of *atg1abct* versus Col-0 plants. Each protein was plotted according to its log_2_ fold change (FC) in abundance (*atg1abct*/Col-0) and its -log_10_ (*p* value) based on three biological replicates. **B**, **C** ATG1 significantly affects plant metabolism and stress response related proteins. GO enrichment of DEPs between Col-0 and *atg1abct*. The color of the dot reflects the *p* value. The size of the dot represents the number of enriched DEPs. BP, biological process. CC, cellular component. MF, molecular function. The fold enrichment = (number of differentially expressed genes with the GO term/number of differentially expressed genes) (number of expressed genes with the GO term/number of expressed genes)

DEPs between the MS_*atg1abct* and MS_Col-0 comparison groups were examined (Table S1). A total of 180 DEPs that are upregulated and 54 that are downregulated (Fig. 3A and S2A). Given the critical role of ATG1 in autophagy, four of DEPs (acyl-CoA binding proteins 3 (ACBP3), SNF1 kinase homolog 10 (KIN10), trafficking protein particle (TRAPP) complexes subunit gene *Trs33*, and plant ubiquitin regulatory X domain containing protein 4 (PUX4)) were found to be involved in autophagy (Fig. 3A). ACBP3 and KIN10 have been identified involved in regulation of autophagy. By attaching to PE, the phospholipid binding protein ACBP3 impairs ATG8 lipidation and indirectly interferes with the formation of autophagosomes, thereby negatively regulating autophagy [31]. Plant autophagy is positively regulated by KIN10, which phosphorylates ATG6 directly and may also influence ATG1 phosphorylation [32, 33]. TRS33 has been shown to influence autophagy in yeast but has only been found to be important in plants for maintaining apical meristem activity and dominance in *Arabidopsis thaliana* [34, 35]. According to its GO annotation, inner membrane anchored PUX4 is involved in autophagosome assembly and has the capacity to disrupt the cell division cycle 48 (CDC48)-dependent proteolytic pathway in plants [36]. PUX7, PUX8, PUX9, and PUX13, four of the 16 *Arabidopsis thaliana* PUXs, communicate with ATG8 through their ubiquitin interaction motif (UIM)-like sequences to attach to autophagosomes and recruit inactive CDC48 for degradation, while the function of PUX4 in autophagy has not been directly studied [37].

**Fig. 4:**
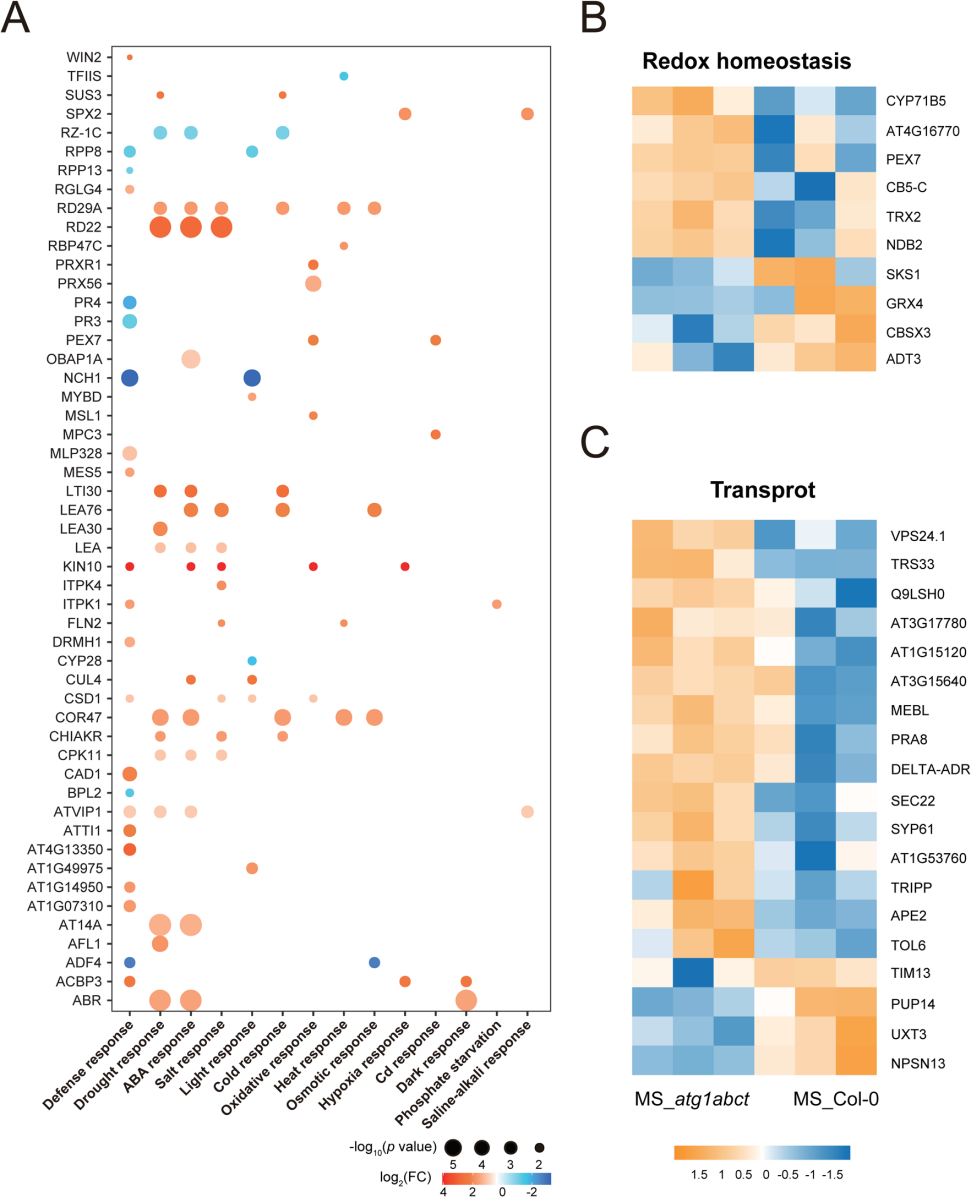
*atg1abct* mutant mainly affects defense and drought response related proteins. **A** The DEPs in the MS_*atg1abct*/MS_Col-0 group were mainly related to the defense and drought response. Each protein was plotted according to its log_2_ fold change (FC) in abundance (MS_*atg1abct*/MS_Col-0) and its -log_10_ (*p* value) based on three biological replicates. The size of the dot reflects the -log_10_ (*p* value). The color of the dot reflects the log_2_ (FC). Heat map of DEPs related to the metabolisms of **B** redox homeostasis, **C** transport. The expression of DEPs in different samples was standardized by *Z*-score method and displayed in different colors in the heat map, where orange represents significantly up-regulated proteins and blue represents significantly down-regulated proteins. The selection criterion for DEPs was l log2 fold change l > 1 and *p* values < 0.05

We also observed that mutations in *atg1abct* mainly resulted in enrichment for GO terms associated with metabolism and various stresses (Fig. 3B, C and Table S2). The primary enrichment to metabolism were inositol phosphate metabolism, glutamine metabolism and fatty acid metabolism. The phosphatidylinositol metabolism-related proteins inositol 1,3,4-trisphosphate 5/6-kinase 1 (ITPK1) and ITPK4, which either directly or indirectly control the levels of inositol pyrophosphate and inositol hexakisphosphate (InsP6) by supplying precursors, were both expressed at higher levels in *atg1abct* [38]. In contrast, phosphate (Pi) homeostasis can be controlled by InsP6 binding to SPX domain-containing protein 2 (SPX2)-PHOSPHATE STARVATION RESPONSE 2 (PHR2) [39]. We suspect that ATG1 is linked to Pi homeostasis because SPX2 proteins connected with Pi starvation are abundantly expressed in *atg1abct* (Fig. 4A and S2B). Six proteins participated in fatty acid metabolism in DEP, three of which (3-ketoacyl-coa synthase 2 (KCS2), long-chain acyl-coenzyme A synthetase 2 (LACS2), ACBP3) were engaged in various stress responses (Table S2). KCS2 takes part in the biosynthesis of very-long-chain fatty acids (VLCFA), and its transcription level is increased in response to salt, abscisic acid (ABA), or drought stress [40]. VLCFAs are precursors for the synthesis of various lipids such as triacylglycerol, sphingolipids, cuticular waxes, and lipid monomers, which play important roles in plant growth and stress response [41]. LACS2 is primarily responsible for polyunsaturated linolenoyl-CoA synthesis. By controlling the permeability of the cuticle surrounding cell, it can improve the tolerance of plants to flooding [42]. ACBP3 can not only affect autophagy by regulating ATG8 stability, but it also influences VLCFA metabolism, allowing plants to respond to hypoxia [43].

In addition, stress-related GO terms, like plant-type hypersensitive response (GO:0009626) and response to water deprivation (GO:0009414), were enriched (Fig. 3B, C). Fifty-one of 234 DEPs connected to stress response, with defense having the highest number of DEPs, followed by drought stress response (Fig. 4A). Consequently, we hypothesized that ATG1 might played a vital role in plant responses to drought and defense. For plants, stress usually induces changes in substance transport and intracellular redox homeostasis. Nine of 19 transport-related DEPs between WT and *atg1abct* were involved in vesicle transport (secretion 22 (SEC22), TRS33, AT3G17780, SYNTAXIN OF PLANTS 61 (SYP61), protein-affected trafficking 4 (PAT4), vacuolar protein sorting 24.1 (VPS24.1), VPS32, prenylated Rab acceptor 8 (PRA8), TRAPP-Interacting Plant Protein (TRIPP)) (Fig. 4C). Six redox homeostasis-related proteins were found to be upregulated in *atg1abct* in comparison to Col-0 (Fig. 4B). 2-Oxoglutarate (2OG) and Fe (II)-dependent oxygenase superfamily protein (AT4G16770), cytochrome P450 71B5 (CYP71B5), cytochrome b5 isoforms C (CYTB5-C) with electron transport function [44], NAD(P)H dehydrogenase B2 (NDB2), and thioredoxins (TRX2) play a role in mitochondria [45, 46] and peroxin 7 (PEX7) that can modulate reactive oxygen species (ROS) metabolism [47]. The expression abundance of four redox homeostasis-related proteins was downregulated in *atg1abct* when compared to Col-0 (Fig. 4B). CBS domain-containing protein (CBSX3) and monothiol glutaredoxin GRXS15 also control ROS in mitochondria [48, 49]. Arogenate dehydratase (ADT3) and multicopper oxidase-like protein skewed 5-similar 1 (SKS1) are responsible for coordinating ROS homeostasis at the chloroplast and cell surface, respectively [50, 51]. In summary, plant responses to stress are greatly impacted by mutations in *atg1abct*.

### ATG1-dependent autophagy affects plant transport and stress response related proteins

**Fig. 5:**
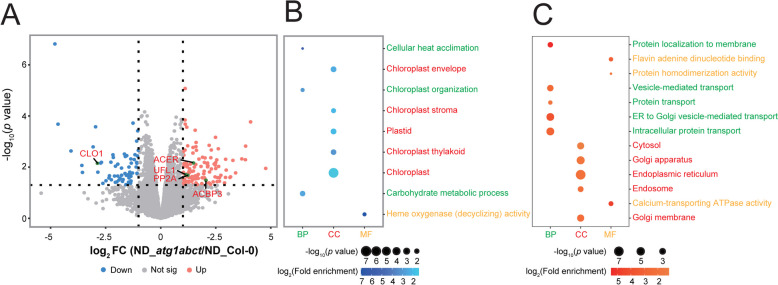
ATG1-dependent autophagy mainly affects transport. **A** Volcano plots of DEPs identified by the proteome analysis in *atg1abct* versus Col-0 plants under ND treatment. Each protein was plotted according to its log_2_ fold change in abundance (*atg1abct*/Col-0) and its -log_10_ (*p* value) based on three biological replicates. **B**, **C** ATG1-dependent autophagy significantly affects plant transport. GO enrichment of DEPs between Col-0 and *atg1abct* under ND treatment. The color of the dot reflects the *p* value. The size of the dot represents the number of enriched DEPs. BP, biological process. CC, cellular component. MF, molecular function. The fold enrichment = (number of differentially expressed genes with the GO term/number of differentially expressed genes) (number of expressed genes with the GO term/number of expressed genes)

Given that ATG1 significantly contributes to the initiation of autophagy in plants under ND, we thus compared the protein profile between ND_*atg1abct* and ND_Col-0. A total of 185 DEPs were obtained, 123 of which were upregulated and 62 of which were downregulated (Fig. S2A). There were five DEPs (ACBP3, protein phosphatase 2A (PP2A), alkaline ceramidase (ACER), ubiquitin-fold modifier 1 E3 ligase 1 (Ufl1), CALEOSIN 1 (CLO1)) have been proved involving with autophagy (Fig. 5A). The upregulation of ACBP3 in both the ND_*atg1abct*/ND_Col-0 and MS_*atg1abct*/MS_Col-0 implies that either the regulation of autophagy by ACBP3 is may related to ATG1 or that ATG1 may regulate fatty acid metabolism through ACBP3. Although PP2A has been shown to negatively control the induction of autophagy in yeast, it has only been observed to obstruct the initial stages of the autophagy pathway in plants; furthermore, it has not been proved that PP2A is directly involved in the ATG-dependent pathway [52, 53]. Upon ND treatment, *atg1abct* showed increased expression of ACER, which is a crucial component of sphingolipid metabolism pathway and played a part in autophagy. This confirms previous findings that autophagy affects sphingolipid homeostasis [54]. Ufl1, which controls endoplasmic reticulum (ER) autophagy, was upregulated in *atg1abct* under ND treatment. And Ufl1 could interact with ATG1, ATG6 and ATG8 [55]. However, CLO1, which was significantly downregulated in ND_*atg1abct*/ND_Col-0, can interact with ATG8 and may be involved in microlipophagy [56].

One hundred and eighty-five DEPs in ND_*atg1abct*/ND_Col-0 was subjected to GO analysis, which revealed that DEPs were mainly enriched in chloroplasts, including the chloroplast envelope, chloroplast stroma, and chloroplast thylakoid (Fig. 5B). This indirectly verified the importance of nitrogen in chloroplasts, which over 70% of available nitrogen is used by plants to keep their chloroplasts functioning [57]. Apart from being the site of photosynthesis, chloroplasts also aid plants in stress adaptation by breaking down and recycling their constituent parts [58]. The autophagy pathway in plants can be triggered by ND treatment to cause chloroplast degradation, but the loss of *atg1abct* blocked the autophagy pathway, resulting in the presence of more chloroplast related proteins.

**Fig. 6:**
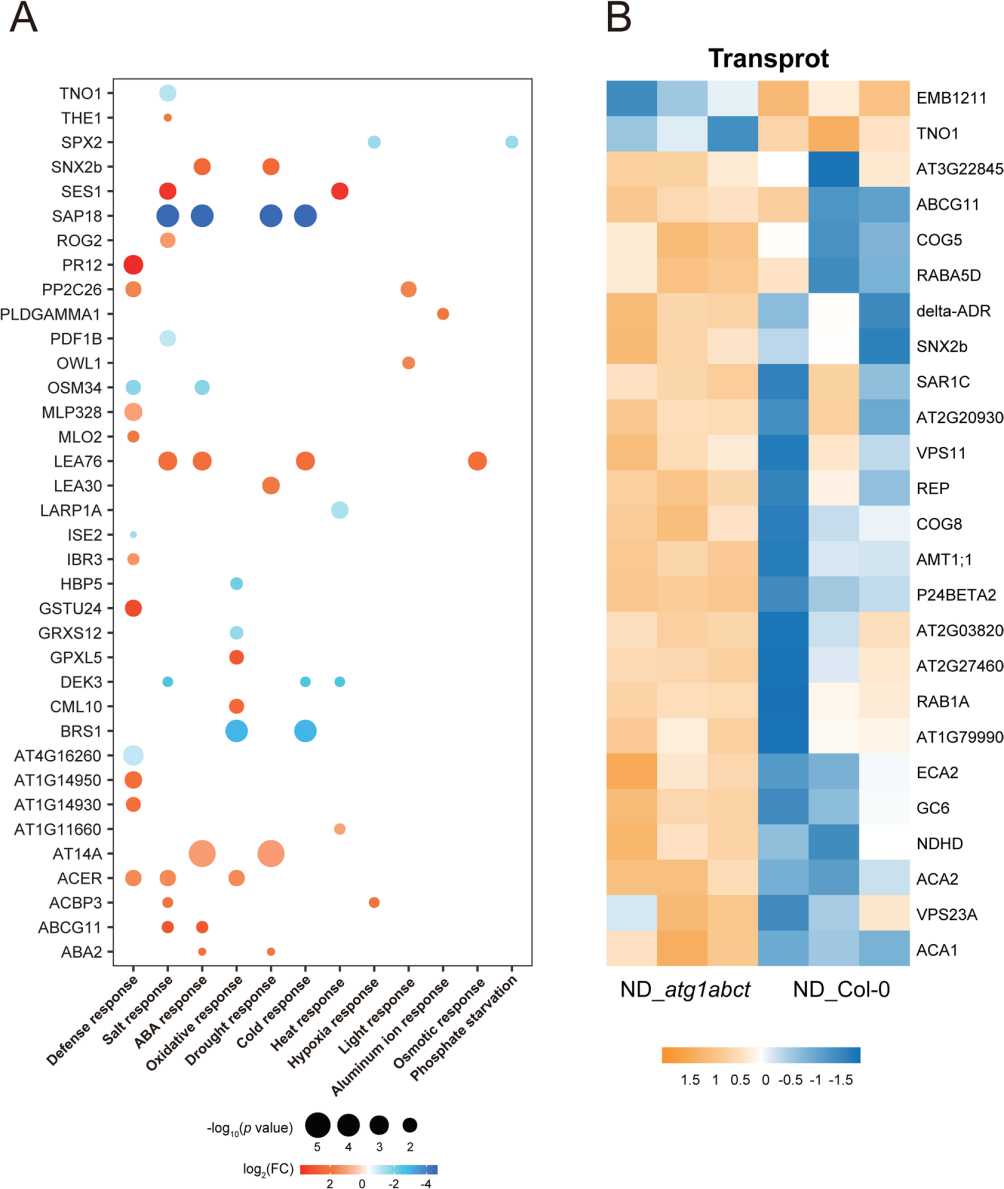
ATG1-dependent autophagy mainly affects defense response and salt response. **A** The DEPs in the ND_*atg1abct*/ND_Col-0 group were mainly related to the defense and salt response. Each protein was plotted according to its log_2_ fold change (FC) in abundance (ND_*atg1abct*/ND_Col-0) and its -log_10_ (*p* value) based on three biological replicates. The size of the dot reflects the -log_10_ (*p* value). The color of the dot reflects the log_2_ (FC). Heat map of DEPs related to the metabolisms of **B** transport. The expression of DEPs in different samples was standardized by *Z*-score method and displayed in different colors in the heat map, where orange represents significantly up-regulated proteins and blue represents significantly down-regulated proteins. The selection criterion for DEPs was l log2 fold change l > 1 and *p* value < 0.05

Given the strong correlation between stress and autophagy, we found that 36 out of 185 DEPs were associated with stress (Fig. 6A). Compared to MS_Col-0, the ND treatment exhibited the highest DEPs ratio linked to defense. Nitrogen plays a complex and critical role in plant defense mechanisms. Under nitrogen deficiency conditions, plant defense responses can be affected by the accumulation and expression of hormones, plant metabolic pathways, and antioxidant enzyme activities [59–62]. Two of the 12 defense-related DEPs are involved in the regulation of ROS responses (MLO2 and OSM24) [63, 64]. Two DEPs (ACER, IBR) are associated with plant hormones and play a role in plant defense [65, 66].

Stress response is closely related to the transport process of substances in plants [67, 68]. We also found significant enrichment of transport-related biological processes in BP (Figs. 5C and 6B). Twelve of the 25 transport-related DEPs were involved in vesicle transport (COG complex subunits (COG8, COG5), Rab GTPase homolog 1A (RAB1A), secretion-linked Ras super family 2 (SAR2), p24 subfamily beta 2 (P24BETA2), vacuolar protein sorting-associated protein 11 (VPS11), Rab escort protein (REP), GOLGIN CANDIDATE 6 (GC6), AT3G22845 AT1G79990, AT2G20930, and AT2G27460). In ND_*atg1abct*/ND_Col-0, three proteins involved in calcium transport (Endomembrane Ca^2+^ ATPase 2 (ECA2), *Arabidopsis thaliana* Ca^2+^-ATPases 1 (ACA1), ACA2) were upregulated, among which ACA1 and ACA2 are crucial for controlling Ca signaling [69]. As a multifunctional messenger, Ca^2+^ is essential in almost all abiotic stresses [70]. Ammonium transporter 1;1 (AMT1;1) encodes a plasma membrane-localized ammonium transporter that helps roots enrich NH^4+^ [71]. In plants, ND resulted in an imbalance in the homeostasis of ammonium, whereas the *atg1abct* mutation blocked autophagy, potentially causing an accumulation of AMT1;1. These analyses show that ATG1-dependent autophagy significantly affects plant responses to stress and material transport.

### ATG1-dependent autophagy affects the expression of phytohormone-related proteins

Phytohormones are vital signaling molecules that are endogenous metabolites of plants and are crucial to the life cycle of plants. It has gradually become apparent how closely autophagy and hormone signaling pathways are related [72]. Our data analysis found 27 and 16 hormone-related DEPs in MS_*atg1abct*/MS_Col-0 and ND_*atg1abct*/ND_Col-0, respectively (Fig. S3, Table S1). In both the ND and control (MS) treatments, DEPs linked to the ABA were the most prevalent. Among hormone-associated DEPs, AT14A was the only one that showed upregulation in both ND_*atg1abct*/ND_Col-0 and MS_*atg1abct*/MS_Col-0. Through controlling protein phosphatase 2C 51 (PP2C51) and ABI5 expression, AT14A contributes to the ABA pathway that improves drought tolerance [73]. It is possible that ATG1 uses AT14A to control the ABA pathway.

At present, the relationship between ABA and autophagy only proves that ABA modulates autophagy, but it is unknown whether autophagy directly adjusts ABA pathway [72]. ATG1-dependent autophagy may regulate ABA when according to our data analysis in this study. Four DEPs (VPS23A, SNX2B, NUCLEAR FACTOR Y SUBUNIT C4 (NF-YC4), and PP2A) that regulated ABA signaling pathway in ND_*atg1abct*/ND_Col-0 [74–77], and one DEPs (ABA2) involved in ABA synthesis (Fig. S3B) [78].

In addition to ABA, we discovered brassinosteroid (BR)-associated DEPs (BR insensitive 1 suppressor 1 (BRS1), THESEUS1 (THE1), and PP2A) in ND_*atg1abct*/ND_Col-0 [79–81]. BR signaling and autophagy pathways interact with each other [72]. The autophagy-related protein PP2A is also beneficial to BR signaling [80]. We also postulated a potential connection between BR signaling via PP2A and autophagy.

### The absence of ATG1 improves tolerance to drought stress in Arabidophsis thaliana

**Fig. 7:**
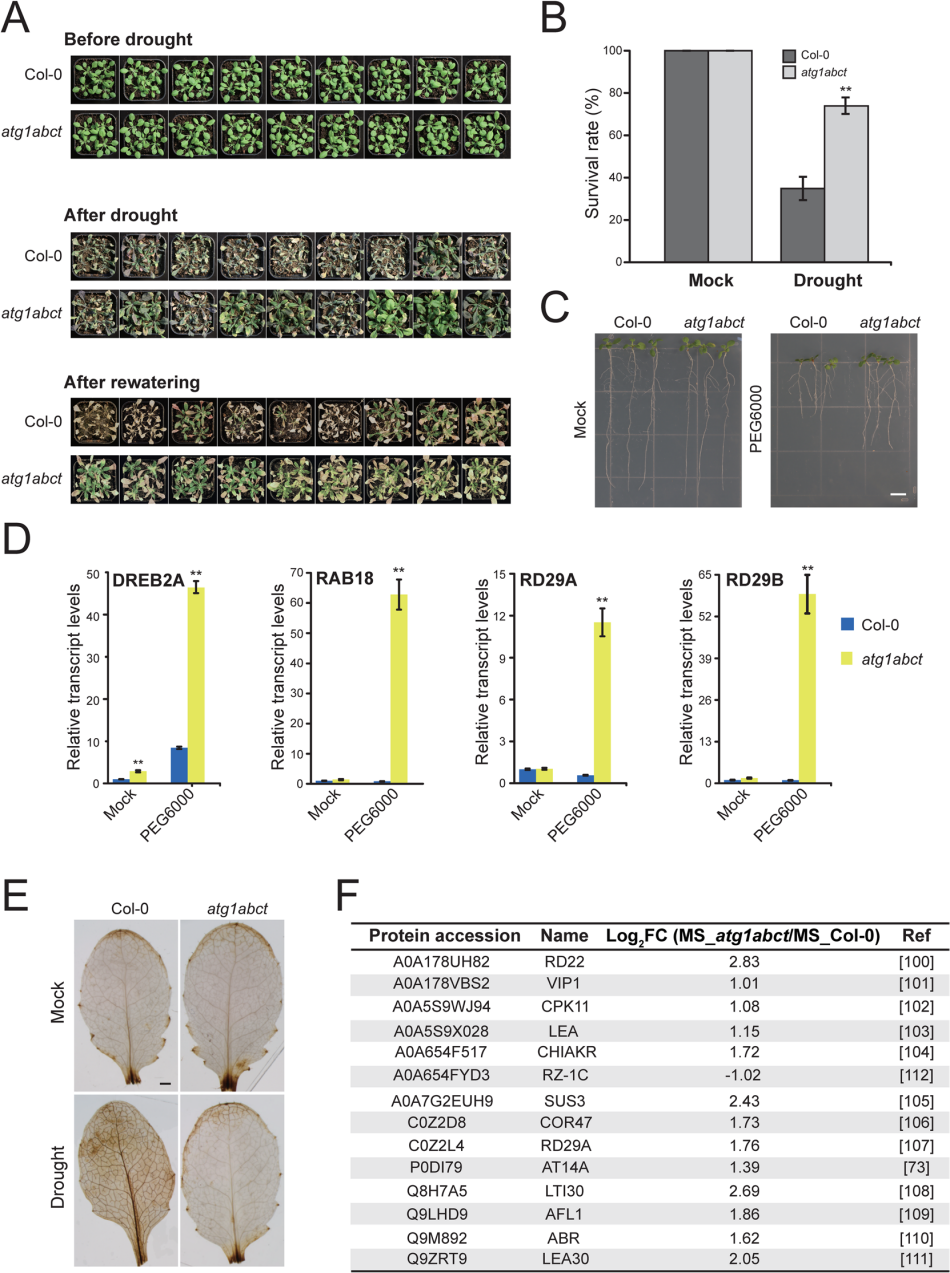
*atg1abct* mutation improves tolerance to drought stress. **A** 20-days-old Col-0 and *atg1abct* plants were subjected to severe drought stress with water deprivation for 12 days and then recovered for 8 days, n = 50. **B** Survival rate of plants after 8 days of recovery. Asterisks indicate significant differences between Col-0 and *atg1abct* mutant revealed by Tukey’s test. ** indicates *p* value < 0.01. **C** Seeds of Col-0 and *atg1abct* were germinated on 1/2 MS medium (control), and 1/2 MS medium containing 6% PEG6000. Representative plants were photographed after 10 days. Scale bar = 0.5 cm. **D** qRT-PCR assay to determine the transcriptional level of drought-responsive marker genes DREB2A, RAB18, RD29A, and RD29B. 10-day-old seedlings were treated with 15% PEG6000 to stimulate drought stress for 1 h. Data are represented as means ± SD of three biological replicates. Asterisks indicate significant differences (** *p* < 0.01) according to Tukey’s test. **E** DAB staining of Col-0 and *atg1abct*. Scale bar = 1 mm. **F** The list of drought related DEPs in MS_*atg1abct*/MS_Col-0. FC, fold change

Given that a large number of DEPs were linked to drought stress responses in the MS_*atg1abct*/MS_Col-0 comparison group, we hypothesized that ATG1 has a significant role in plants under drought stress (Fig. 4A). Thus, drought survival assays were conducted. Following a period of drought and rewatering, *atg1abct* exhibited a significantly higher survival rate compared to Col-0 (Fig. 7A, B). To learn more about how Col-0 and *atg1abct* early-stage seedlings react to drought conditions, they were subjected to Polyethylene glycol (PEG) 6000 to mimic drought stress. The findings demonstrated that the root of *atg1abct* were longer than those of Col-0 (Fig. 7C). This indicated that *atg1abct* seedlings grew better in response to PEG6000 and had a similar phenotype to adult plants. After PEG stress processing, 7-day-old seedlings were subjected to qRT-PCR. The results found that *atg1abct* plant drought stress related gene expression is significantly higher than Col-0 (Fig. 7D). Given the strong association between ABA content and drought tolerance of plants, we examined endogenous ABA content in seedlings treated with PEG6000. After PEG6000 treatment for 4 h, the ABA content in *atg1abct* mutants was significant higher than Col-0 seedlings (Fig. S4). These findings imply that a vital role of ATG1 in drought stress.

To investigate whether *atg1abct* affects ROS accumulation and scavenging under drought stress, plant leaves were stained with 3,3′-diaminobenzidine (DAB). After drought treatment, *atg1abct* showed lighter leaf color than Col-0, suggesting that Col-0 accumulated larger amounts of ROS (Fig. 7E). In conclusion, mutation of ATG1 can provide *Arabidopsis thaliana* drought tolerance and reduce ROS accumulation.

## Discussion

In the past decade, due to its critical role in mediating plant growth and development as well as responding to biotic and abiotic stresses, autophagy has become the hotspot of plant research [30, 82, 83]. As one of the most upstream elements of autophagy, ATG1’s role must be fully comprehended for the purpose to advance our knowledge of the mechanism governing plant autophagy regulation. In this study, we quantitated 5185 *Arabidopsis thaliana* proteins through proteomics assay. Several autophagy related proteins were validated in this work, and new pathways and possible mechanisms for ATG1-dependent autophagy and ATG1 function in addition to managing autophagy were also identified.

### ATG1-dependent autophagy affects ER homeostasis

Ufmylation is a unique post-translational modification that is highly conserved in plants and mammals. It is mediated by the ubiquitin-like protein ubiquitin folding modifier 1 (Ufm1), which serves a variety of purposes in various cellular processes [84–86]. Ufl1, an E3 ligase of the ufmylation system, was identified in this study. Previous study shown that, under salt stress, Ufl1 is drawn to the ER and engages in direct interaction with ATG1, ATG6, and ATG8. This functions as a link between the ER sheets that require degradation and the production of autophagosomes, facilitating the removal of accumulated ER through reticular phagocytosis and restoring cellular homeostasis [87]. Both at ND_*atg1abct*/ND_Col-0 and ND_*atg1abct*/MS_*atg1abct*, Ufl1 was upregulated. Given that ER stress is induced by salt stress in *Arabidopsis thaliana*, ND may similarly cause ER stress and increase the expression of Ufl1. But the absence of *atg1abct* prevents autophagy and makes it impossible to recycle ER sheets, which causes Ufl1 protein to build up. This means that ND stress also might trigger ER expansion and maintain ER homeostasis via autophagy, besides salt stress.

In addition, we observed that two ER-related proteins (Der1 and AtERdj2B) were upregulated in the ND_*atg1abct*/ND_Col-0 comparison group. Derlin-1 (Der 1), as a component of the larger complex in the ER membrane, may facilitate the recognition and reverse translocation of certain misfolded proteins from the ER to the cytoplasm in the protein quality control pathway [88]. ERdj2B is an integral ER membrane protein, rendering its J domain facing the ER lumen [89]. Therefore, we hypothesize that ATG1-dependent autophagy may also affect ER homeostasis through other pathways.

### ATG1-dependent autophagy regulating the plant hormone ABA

Plants can maintain cell stability by triggering hormones in response to stress, including ABA mediated drought [90]. In this study, several DEPs were found to be involved with ABA in this study (Fig. 7). As a plant growth regulator, the plant hormone ABA can adjust a variety of plant physiological processes. Especially under stress, ABA strengthens plant stress resistance through signal transduction and control of genes or proteins expression [91]. Although there is a strong correlation between autophagy and ABA, it has only been discovered that ABA regulates autophagy; it has not been established whether autophagy directly regulates ABA pathway [72]. Our omics analysis revealed that ABA2 was raised in ND_*atg1abct*/ND_Col-0, which was associated with ABA synthesis. This implies that autophagy may regulate ABA through ABA2. Moreover, ND_*atg1abct*/ND_Col-0 also contained four DEPs that controlled ABA signaling. Among these, SNX2b acts in the vesicular transport of endosomes to vacuoles, which can take charge of the amount of ABA in cells and, in turn, affect stomatal motility and drought tolerance [75]. Given that SNX2b was upregulated in ND_*atg1abct*/ND_Col-0, and downregulated in ND_Col-0/MS_Col-0, it is possible that SNX2b may be degraded by autophagy. This provides evidence that autophagy may control ABA by splicing SNX2b. Additionally, PP2A was upregulated in ND_*atg1abct*/ND_Col-0 and downregulated in ND_Col-0/MS_Col-0. ABA-activated SnRK2-type protein kinases interact with multiple PP2A subunits [92]. Besides, there is a novel branch of ABA signaling involving the pyrabactin resistance (PYR)/PYR1-like (PYL)/regulatory components of the ABA receptor (RCAR) receptor-PP2A complex that occurs between ABA and PP2A. Through the manipulation of auxin transport, this signaling pathway modulates the formation of lateral roots and root geotropism [77]. Therefore, it is also possible that autophagy regulates ABA by way of PP2A. These ABA-related DEPs offer new perspectives on the future directions of autophagy and ABA research.

### ATG1 is associated with inositol phosphate metabolism

Inositol phosphates are a type of small molecule messengers that affects cellular physiology and is crucial in cellular decision-making [93]. Inositol phosphates comprise two major classes: lipid-anchored phosphatidylinositol phosphates (PtdInsPs) and soluble inositol phosphates (InsPs). Among the InsPs, InsP6 is the most abundant species in a bulk of organisms and cell lines, which can chelate metal ions and take part in signal transduction in plant cells [94, 95]. ITPK1 and ITPK4, coupled with InsP6, showed higher abundance in MS_*atg1abct*/MS_Col-0. ITPK1 is in responsible for catalyzing the phosphorylation of InsP6 into symmetrical 5-InsP7 [96]. ITPK4 is the key enzyme to produce InsP4 precursors for InsP6 biosynthesis [97]. In eukaryotes, there is a strong relationship between inositol phosphate metabolism (such as InsP6 synthesis) and intracellular Pi homeostasis. A key player in Pi homeostasis, ITPK1 is also necessary for InsP6 synthesis in Pi-sufficient environments [97]. The correlation between Pi homeostasis and autophagy is likewise complicated and poorly understood. It has been established that both basal autophagy and phosphorus starvation-induced autophagy maintain phosphorus homeostasis by regulating the expression of phosphate transporter 1 (PHT1) [98]. Only MS_*atg1abct*/MS_Col-0 showed up-regulation of ITPK1 and ITPK4, and there was no change in protein expression following ND treatment. So, we postulated that ATG1 might impact Pi homeostasis and phosphatidylinositol metabolism by ITPK1 and ITPK4.

### ATG1 is involved in fatty acid metabolism

Long-chain acyl-CoA synthetase (LACS) is a class of enzymes belonging to the ACS family that plays important roles in fatty acid synthesis and catabolism [99]. The LACS gene family has nine members (LACS1-9) in the model plant *Arabidopsis thaliana*, each of which has a distinct function in lipid metabolism [100]. In this study, LACS2 and LACS8 were quantified. LACS2 can efficiently catalyze the monomeric ω-hydroxypalmitic acid and is mainly involved in the synthesis of cutin [101]. LACS8 exhibits the highest level of expression in embryos and shares a significant degree of sequence similarity with LACS9 [102]. Based on the up-regulation of LACS2 and LACS8 at MS_*atg1abct*//MS_Col-0 and ND_*atg1abct*/ND_Col-0, it can be concluded that variations in their expression levels were exclusively connected to ATG1. In other words, ATG1 may affect lipid metabolism. In Chlamydomonas, LACS1 and LACS2 are localized in the ER and/or lipid droplets, and they are both involved in activating fatty acids for ER lipid synthesis under normal growth conditions. LACS2 is activated in response to stressors like ND for the purpose to supply enough acyl CoA for acyltransferases on the ER membrane to synthesize TAG, hence preventing the impact on membrane lipid synthesis [103]. In this work, LACS2 and LACS8 were both upregulated at MS_*atg1abct*/MS_Col-0 and ND_*atg1abct*/ND_Col-0, indicating that changes in their expression levels were exclusively correlated to ATG1. We proposed that ATG1 might have an influence on the ER membrane lipid synthesis in *Arabidopsis thaliana* cells.

In addition, ACBP3 was also upregulated in both MS_*atg1abct*/MS_Col-0 and ND_*atg1abct*/ND_Col-0. ACBP3 belongs to the acyl-CoA binding protein family. ACBPs are a conserved family of lipid-binding proteins that are encoded by a family of six genes (ACBP1-6) in *Arabidopsis thaliana* [104]. ACBP3 contains ACB domain and N-terminal transmembrane domain that coincides with the extracellular targeting signal peptide [31]. ACBP3 not only adjusts membrane phospholipid metabolism and ATG8 stability, and plays a function in SA-dependent plant defense signal transduction, but it also participates in plant response to hypoxic stress by regulating VLCFA metabolism [31, 105]. Like LACS2 and LACS8, ACBP3 was increased in both ND and control treatments, indicating that ATG1 was the only factor influencing its expression level change. Through modulation of cell-to-cell cuticle permeability, LACS2 can enhance plants’ ability to withstand flooding [42]. ACBP3 has also been linked to a variety of stress responses. This clearly implies that ATG1 may play a role in the response to various stresses by regulating fatty acid metabolism.

### ATG1 reduces drought tolerance in Arabidopsis

Based on the proteomic data, 14 DEPs related to drought response were identified in MS_*atg1abct*/MS_Col-0 (Fig. 7F). Thirteen drought tolerance related DEPs, including RESPONSIVE TO DESICCATION 22 (RD22), VirE2-interacting protein1 (VIP1), Ca^2+^-dependent protein kinase11 (CPK11), late embryogenesis abundant (LEA), CHLOROPLASTIC ALDO–KETO REDUCTASE (CHIAKR), sucrose synthase 3 (SUS3), COLD-REGULATED 47 (COR47), RESPONSIVE TO DESICCATION 29A (RD29A), AT14A, Low Temperature-Induced 30 (LTI30), AT14A-LIKE1 (AFL1), ABA-RESPONSE PROTEIN (ABR), and late embryogenesis abundant 30 (LEA30), were up-regulated [73, 106–117]. RZ-1C is the down-regulated DEP in MS_*atg1abct*/MS_Col-0. RZ-1c responded more significantly at the initial stage of drought stress, but its effect might be weakened under persistent drought conditions [118]. It is widely known that ABA plays a crucial role in plant responses to drought stress [119]. Proteomic analysis showed that 13 DEPs were related to ABA response in MS_*atg1abct*/MS_Col-0, of which 11 DEPs were also related to drought response (Fig. S3A). This suggests that the mutation of *atg1abct* may enhanced drought tolerant through a pathway associated with ABA. However, through our proteomics data and qPCR results, we found that the expression levels of several genes/proteins from both ABA-dependent (e.g., RD22, RAB18, RD29A/29B, COR47, LTI30) and ABA-independent (DREB2A, RD29A/29B) signaling pathways in *atg1abct* were significantly higher than Col-0, which gave us a hint that *atg1abct* enhance *Arabidopsis* drought tolerance through both ABA-dependent and ABA-independent signal transduction pathways.

Under drought stress, plants also activate antioxidant enzymes to remove ROS and protect cells from oxidative stress damage [120]. PEX7, which controls ROS metabolism, was up-regulated in MS_*atg1abct*/MS_Col-0 [121]. Less accumulation of ROS in *atg1abct* mutants was also observed by DAB staining after drought treatment (Fig. 7E).

## Materials and methods

### Plant growth and nitrogen deficiency treatment

*Arabidopsis thaliana* seeds were sterilized separately in a 0.03% sodium hypochlorite solution, followed by distilled water washing three times, and then stored under continuous darkness at 4 °C for 2 days to induce uniform germination. After that, *Arabidopsis thaliana* seeds were cultivated in liquid MS medium supplemented with 2% (w/v) sucrose (pH 5.7) for 7 days. Seedlings were transferred to either liquid MS medium or nitrogen-deficient (ND) liquid MS medium for additional 2 days, respectively. Seedlings were grown at 21 °C under a long-day photoperiod (16 h light/8 h dark) with 100 μmol/m^2^/s LED lights. Three independent biological replicates of each group were harvested and frozen in liquid nitrogen, ground to a fine powder, and stored at – 80 °C for subsequent analysis.

### Total protein extraction and digestion

Proteins were extracted from each sample with lysis buffer consisting of 4% (w/v) sodium dodecyl sulfate (SDS) and 100 mM Tris–HCl at pH 7.6. Protein concentration estimation was performed using the BCA Protein Assay Kit (Bio-Rad, USA). A quantity of 20 μg total protein per sample was mixed with 5 × loading buffer and boiled for 5 min before being separated by Sodium dodecyl sulfate–polyacrylamide gel electrophoresis (SDS-PAGE) subsequently stained with Coomassie Blue R250. Protein digestion by trypsin was processed using the filter-aided sample preparation (FASP) method described by Wiśniewski et al. [122]. In short, before being transferred to filters, dithiothreitol (DTT) and iodoacetamide (IAA) were added sequentially to the protein sample at 10 mM and 20 mM final concentrations, respectively. The filter was washed thrice with 100 μL UA buffer and twice with 100 μL 25 mM NH4HCO3 buffer. Finally, trypsin was added at a ratio of 1:50 relative to total protein mass, and the sample was incubated at 37 °C overnight. Collected peptides were desalted on C18 Cartridges (Empore™ SPE Cartridges C18 (standard density), bed I.D. 7 mm, volume 3 mL, Sigma), then concentrated by vacuum centrifugation and resuspended in 40 μL 0.1% formic acid. Absorbance measured at 280 nm (A280) was used to calculate peptide concentration.

### Liquid chromatography-tandem mass spectrometry analysis

Liquid chromatography-tandem mass spectrometry (LC–MS/MS) of tryptic peptides was analyzed using a timsTOF Pro mass spectrometer coupled with a nanoElute (both Bruker, USA). Resuspended peptides (0.4 μg) were loaded onto a C18 reversed-phase analytical column (75 μm × 25 cm, 1.9 μm resin, Thermo Scientific Easy Column) in 95% solvent A. Analytical separation was performed using a linear gradient from 5 to 40% solvent B over 48 min followed by an increase to 90% B over 3 min and hold at 90% B for 10 min. The flow rate was maintained at 300 nL/min. Solvents A and B were water with 0.1% formic acid and acetonitrile with 0.1% formic acid, respectively. MS data was acquired with the m/z range from 100 to 1700. The mass spectrometer was operated in parallel accumulation serial fragmentation (PASEF) mode and sampled an ion mobility ranges from 1/K0 = 0.6 to 1.6 Vs/cm^2^. One MS1 acquisition was followed by 10 PASEF mode acquisitions of MS2. The dynamic exclusion time of the MS2 scan was set to 24 s to avoid duplicated scans of the parent ion.

### Sequence database search

Database search was performed with the MaxQuant platform, version 1.6.14.0 (http://www.maxquant.org) [123], employing the Andromeda search engine [124] against the *A. thaliana* UniProt FASTA database (October 2022). Carbamidomethyl (Cys), acetylation (protein N-terminal) and oxidation (Met) were selected as fixed and variable modifications, respectively. Trypsin/P was used as the digesting enzyme with up to two missed cleavages. Peptide mass tolerance was set to 6 ppm during the main search, and fragment mass tolerance was set to 20 ppm. The “Unique + razor” was selected for protein ratio calculation. The false discovery rates (FDR) of peptide and protein were set to 0.01 based on a target-decoy reverse database. “Match between runs” was enabled to improve proteome coverage with a match time window of 2 min. Label-free quantitation (LFQ) was conducted using the MaxLFQ algorithm built into MaxQuant for protein quantification [125].

### Bioinformatic analysis

The “MaxQuant” quantification file “proteinGroups.txt” was loaded and analyzed using the R package Differential Enhancement of Proteomics data (DEP) [126]. Reverse, potential contaminant, and “only identified by site” identifications in the protein list were discarded. Protein entries whose LFQ intensity values in at least one set of biological replicates were all identified could be retained. LFQ intensities were log2 transformed and normalized using the variance-stabilizing normalization (VSN) method [127]. The missing values were replaced with random values obtained from a median downshifted Gaussian distribution performed separately for each group from a distribution with a width of 0.3 and a downshift of 1.8.

Principal component analysis (PCA) for proteomics data was computed using the prcomp function in R to assess the reproducibility of the biological replicates. The criteria for selecting differentially expression proteins (DEPs) were based on *p*-value < 0.05 and l log2 fold change l > 1. Proteins with similar functions were grouped by k-means clustering analysis of DEPs. CELLO (http://cello.life.nctu.edu.tw/) was used for predicting protein subcellular localization. Gene Ontology (GO) term enrichment analysis was done by DAVID [128, 129]. The R Studio Software (version 4.3.1) was used to perform data visualizations.

### Drought phenotype analysis

The PEG-infused plates were prepared as described by Verslues et al. [130]. An overlay solution containing PEG was poured over 1/2 MS agar plates, and PEG was allowed to diffuse into the medium. Seeds were sown on 1/2 MS media supplemented with or without 6% (w/v) PEG6000, and pictures were taken after 10 days.

Plants were grown in soil under long-day conditions (16 h light/8 h dark) at 21 °C for 2 weeks and subjected to drought stress by ceasing watering for 12–15 days. In the experiment, a plastic cup with 350 g soil (1:2 of black soil/vermiculite) was planted with at least 50 plants under long-day conditions. The plants were re-watered when significant differences in wilting were observed. Surviving plants were counted after eight days of re-watering. Each experiment was repeated at least three times.

### RT-PCR assay

Ten-day-old seedlings grown on 1/2 MS solid plates under continuous white were treated with 15% polyethylene glycol (PEG) 6000 to mimic drought stress for 1 h. Total RNA was extracted from the samples using an Ultrapure RNA Kit (CWBio) and was subjected to DNA removal and reverse transcription using a FastQuant RT kit with gDNase (TIANGEN). qRT–PCR were performed using ACTIN 2 as internal controls. qPCR was carried out using SsoFast EvaGreen Supermix (Bio-Rad) and a CFX96 Touch Real-Time PCR Detection System (Bio-Rad) as recommended by the manufacturer. The primers used for qRT–PCR are listed in Supplemental Table S3.

### Physiological measurements

The *Arabidopsis thaliana* leaves were selected for DAB chemical staining analysis after 9 days of drought treatment, and plants grown in normal conditions were used as control. Hydrogen peroxide accumulation was detected by DAB staining. Leaves were placed in 1 mg/mL DAB solution (1 mg/ml DAB, 200 mM Na_2_HPO_4_ and 0.05% v/v Tween 20, PH 5.5–6.0) and incubated at 24 °C for 2 h in the dark. Acetic acid to glycerol to ethanol mixture (volume ratio 1:1:3) was used to remove chlorophyll. The accumulation of hydrogen peroxide was observed under a stereo microscope.

### Abscisic acid content determination

Fourteen-day-old seedling were treated with 20% PEG6000 for 0 h and 4 h. Samples were ground to powder with liquid nitrogen, 0.1 g of tissue was added to 0.9 ml of PBS (PH = 7.2), and the supernatant was collected by centrifugation at 2500 g for 20 min after completion of full homogenization. The plant hormone abscisic acid (ABA) ELISA kit was used to detect the abscisic acid content of the samples.

## Conclusions

Our work highlighted that ATG1-dependent autophagy can regulate multiple biological processes through protein quality control in plants. Together with previous findings that autophagy adjusts sphingolipid homeostasis and plant immunity [54, 131]. Here, we found that (1) ND stress may also induce ER expansion, and then autophagy is associated with ER homeostasis; (2) autophagy may regulate ABA through ABA synthesis, vesicular trafficking, and even PP2A-related signaling pathways; (3) ATG1 affects phosphatidylinositol metabolism; (4) ATG1 may participate in the response to stress by affecting fatty acid metabolism. These proteomic analyses of ATG1 and ATG1-dependent autophagy unveil a comprehensive plant ATG1 signaling network and give novel directions for the potential regulatory mechanisms of autophagy in plants.

## Supplementary Information

Supplementary Material 1: Table S1. K-means cluster analysis and GO enrichment analysis of DEPs were performed; Table S2. Differentially abundant proteins; Table S3. Primer for qPCR analysis; Fig. S1. Most DEPs are located in the cytoplasm and nucleus; Fig. S2. Proteomics analysis of DEPs among the different groups; Fig. S3. Comprehensive analysis of the influence of *atg1abct* on the expression of hormone-related proteins. Fig. S4. Analysis of ABA content in Col-0 and *atg1abct* mutant. 14-day-old Col-0 and *atg1abct* were treated with 20% PEG6000 for 0 h and 4 h, respectively. The error bars represent mean ± SD (*n* = 3). Significant differences were determined using Student’s *t*-test: *p < 0.05.

## Data Availability

The data presented in this study are available upon request from the corresponding author. The mass spectrometry proteomics data have been deposited in ProteomeXchange (http://proteomecentral.proteomexchange.org/cgi/GetDataset) under PX ID PXD057141.

## Abbreviations

ATG: Autophagy-related
PI3K: Phosphatidylinositol 3-kinase
PI3P: Phosphatidylinositol-3-phosphate
PE: Phosphatidylethanolamine
TOR: Target of rapamycin
TRAF: Tumor necrosis factor receptor-associated factor
SINAT: SEVEN IN ABSENTIA OF *ARABIDOPSIS* THALIANA
SnRK1: Sucrose non-fermenting-1 (SNF1)-related protein kinase 1
ULK1: Unc-51 like kinase-1
LC–MS/MS: Liquid chromatography-tandem mass spectrometry
ND: Nitrogen deprivation
ER: Endoplasmic reticulum
GO: Gene Ontology
PCA: Principal component analysis
DEPs: Differentially expressed proteins
ACBP3: Acyl-CoA binding proteins 3
KIN10: SNF1 kinase homolog 10
TRAPP: Trafficking protein particle
PUX: Plant ubiquitin regulatory X domain containing protein
CDC48: Cell division cycle 48
FC: Fold change
BP: Biological process
CC: Cellular component
MF: Molecular function
ITPK: Inositol 1,3,4-trisphosphate 5/6-kinase
InsP6: Inositol hexakisphosphate
KCS2: 3-Ketoacyl-coa synthase 2
LACS: Long-chain acyl-coenzyme A synthetase
VLCFAs: Very-long-chain fatty acids
ABA: Abscisic acid
SEC22: Secretion 22
SYP61: SYNTAXIN OF PLANTS 61
PAT4: Protein-affected trafficking 4
VPS: Vacuolar protein sorting
PRA8: Prenylated Rab acceptor 8
TRIPP: TRAPP-Interacting Plant Protein
CYP71B5: Cytochrome P450 71B5
CYTB5-C: Cytochrome b5 isoforms C
NDB2: NAD(P)H dehydrogenase B2
PEX7: Peroxin 7
ADT: Arogenate dehydratase
PP2A: Protein phosphatase 2A
ACER: Alkaline ceramidase
Ufl1: Ubiquitin-fold modifier 1 E3 ligase 1
CLO1: CALEOSIN 1
RAB1A: Rab GTPase homolog 1A
SAR2: Secretion-linked Ras super family 2
P24BETA2: P24 subfamily beta 2
REP: Rab escort protein
GC6: GOLGIN CANDIDATE 6
ECA2: Endomembrane Ca^2+^ ATPase 2
ACA: *Arabidopsis thaliana* Ca^2+^-ATPases
AMT1;1: Ammonium transporter 1;1
NF-YC4: NUCLEAR FACTOR Y SUBUNIT C4
BR: Brassinosteroid
BRS1: BR insensitive 1 suppressor 1
THE1: THESEUS1
PEG: Polyethylene glycol
DAB: 3,3′-Diaminobenzidine
Ufm1: Ubiquitin folding modifier 1
Der1: Derlin-1
ROS: Reactive oxygen species
PtdInsPs: Phosphatidylinositol phosphates
PHT1: Phosphate transporter 1
RD: RESPONSIVE TO DESICCATION
CPK: Ca^2+^-dependent protein kinase
LEA: Late embryogenesis abundant
CHIAKR: CHLOROPLASTIC ALDO–KETO REDUCTASE
SUS3: Sucrose synthase 3
COR47: COLD-REGULATED 47
LTI30: Low Temperature-Induced 30
ABR: ABA-RESPONSE PROTEIN
SDS: Sodium dodecyl sulfate
SDS-PAGE: Sodium dodecyl sulfate–polyacrylamide gel electrophoresis
FASP: Filter-aided sample preparation
DTT: Dithiothreitol
IAA: Iodoacetamide
PASEF: Parallel accumulation serial fragmentation
LFQ: Label-free quantitation
VSN: Variance-stabilizing normalization
