# Supplementary material for: Proteomics revealed novel functions and drought tolerance of Arabidopsis thaliana protein kinase ATG1
Source: BMC Biol. 2025 Feb 21;23:48. doi: 10.1186/s12915-025-02149-3 (PMC11846238; doi:10.1186/s12915-025-02149-3)
Supplement: Supplementary file 1 — Supplementary Material 1: Table S1. K-means cluster analysis and GO enrichment analysis of DEPs were performed; Table S2. Differentially abundant proteins; Table S3. Primer for qPCR analysis; Fig. S1. Most DEPs are located in the cytoplasm and nucleus; Fig. S2. Proteomics analysis of DEPs among the different groups; Fig. S3. Comprehensive analysis of the influence of atg1abct on the expression of hormone-related proteins. Fig. S4. Analysis of ABA content in Col-0 and atg1abct mutant. 14-day-old Col-0 and atg1abct were treated with 20% PEG6000 for 0 h and 4 h, respectively. The error bars represent mean ± SD (n = 3). Significant differences were determined using Student’s t-test: *p < 0.05. [file 12915_2025_2149_MOESM1_ESM.zip › supplement information-revised-20250131.docx]

Supporting information to: **Proteomics revealed novel functions and drought tolerance of *Arabidopsis* *thaliana* protein kinase ATG1**

Shan Cheng ^a,1^, Siqi Fan ^a,1^, Chao Yang ^a,b^, Weiming Hu ^a,^* and Fen Liu ^a^^,^*

^a^*Lushan Botanical Garden, Jiangxi Province and Chinese Academy of Sciences, Jiujiang 332900, Jiangxi, China*

^b^*College of Life Science, Nanchang University, Nanchang 330031, Jiangxi, China*

^1^These authors contributed equally to this work.

*Corresponding authors.

*E-mail addresses:* huwm@lsbg.cn (W. Hu); liuf@lsbg.cn (F. Liu). Dr. Liu is fully responsible for the distribution of the materials associated with this article.

The following Supporting Information is available for this article:

**Supporting Information**

**Figure S1.** Most DEPs are located in the cytoplasm and nucleus. Subcellular localization of the DEPs in MS_*atg1abct*/MS_Col-0, ND_*atg1abct*/ND_Col-0, ND_*atg1abct*/MS_*atg1abct* and ND_Col-0/MS_Col-0 comparison group.

**Figure S2.** Proteomics analysis of DEPs among the different groups. **A** Number of DEPs between each group. **B** Venn diagram showing unique and overlapping DEPs between the MS_*atg1abct*/MS_Col-0 and ND_*atg1abct*/ND_Col-0. Three overlapping proteins were listed.

**Figure S3.** Comprehensive analysis of the inﬂuence of *atg1abct* on the expression of hormone-related proteins. The histogram shows expression alteration of the up- or down-regulated DEPs associated with hormones in MS_*atg1abct*/MS_Col-0 and ND_*atg1abct*/ND_Col-0.

**Figure S4:** Analysis of ABA content in Col-0 and *atg1abct* mutant.

**Table S1.** K-means cluster analysis and GO enrichment analysis of DEPs were performed.

**Table S2.** Differentially abundant proteins.

**Table S3.** Primer for qPCR analysis.


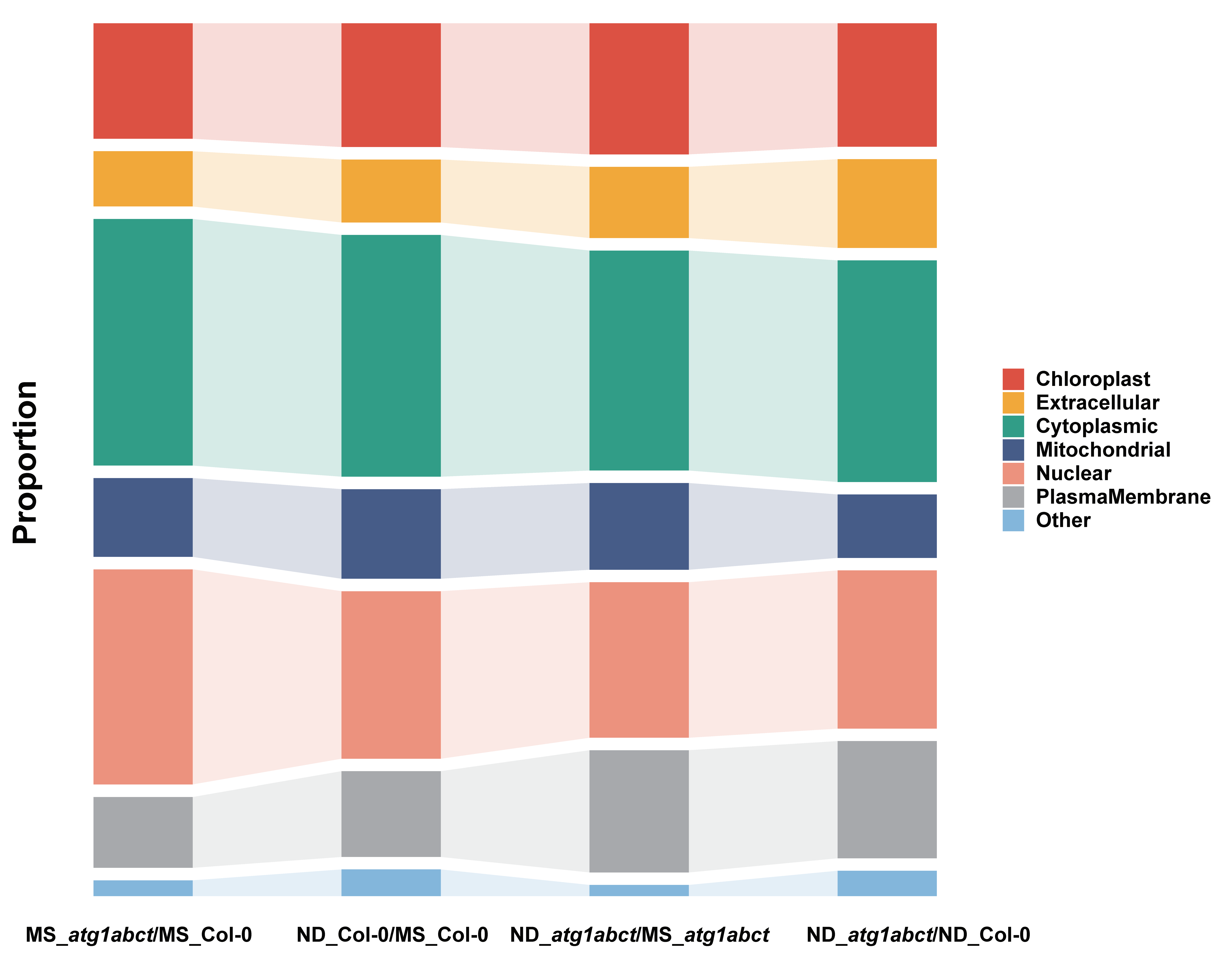


**Figure S1.** Most DEPs are located in the cytoplasm and nucleus. Subcellular localization of the DEPs in MS_*atg1abct*/MS_Col-0, ND_*atg1abct*/ND_Col-0, ND_*atg1abct*/MS_*atg1abct* and ND_Col-0/MS_Col-0 comparison group.

**
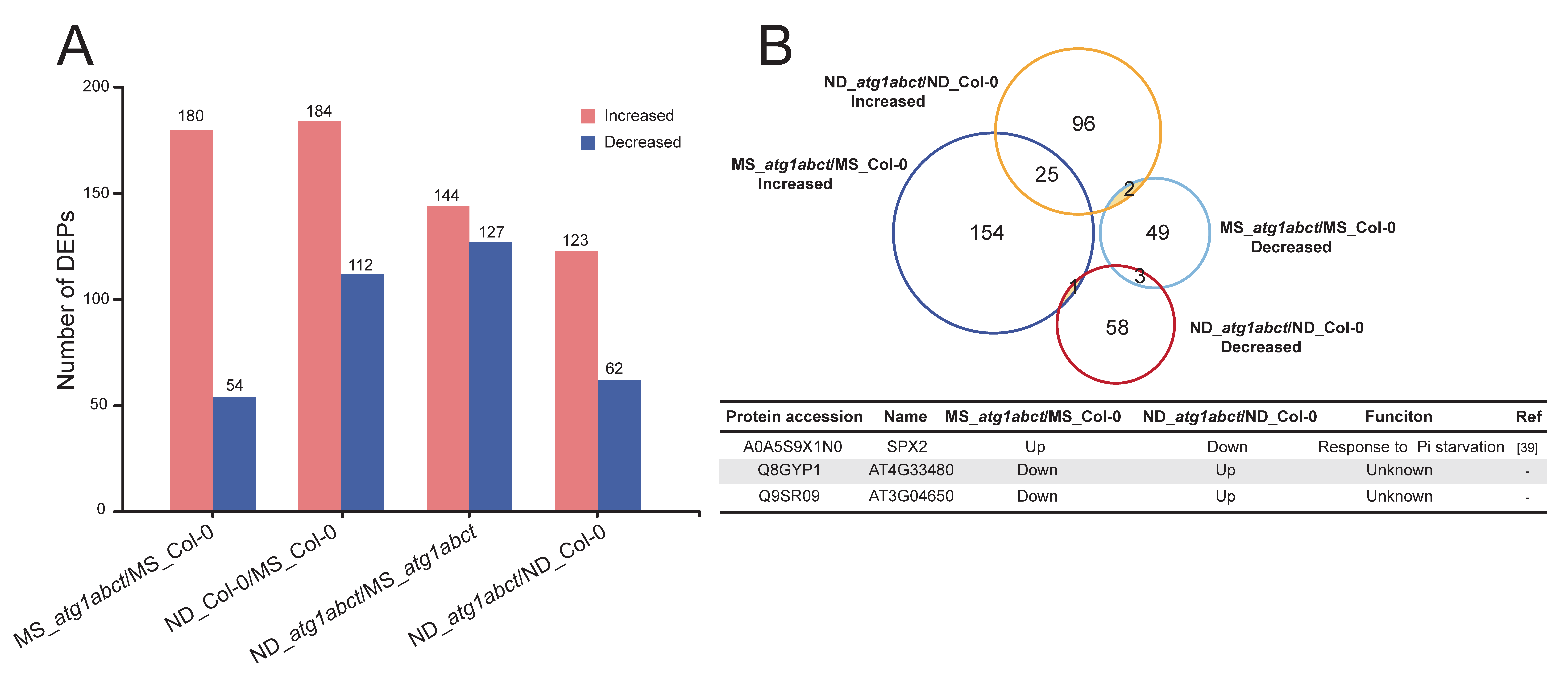
**

**Figure S2.** Proteomics analysis of DEPs among the different groups. **A** Number of DEPs between each group. **B** Venn diagram showing unique and overlapping DEPs between the MS_*atg1abct*/MS_Col-0 and ND_*atg1abct*/ND_Col-0. Three overlapping proteins were listed.





**Figure S3.** Comprehensive analysis of the inﬂuence of atg1abct on the expression of hormone-related proteins. The histogram shows expression alteration of the up- or down-regulated DEPs associated with hormones in MS_*atg1abct*/MS_Col-0 and ND_*atg1abct*/ND_Col-0.


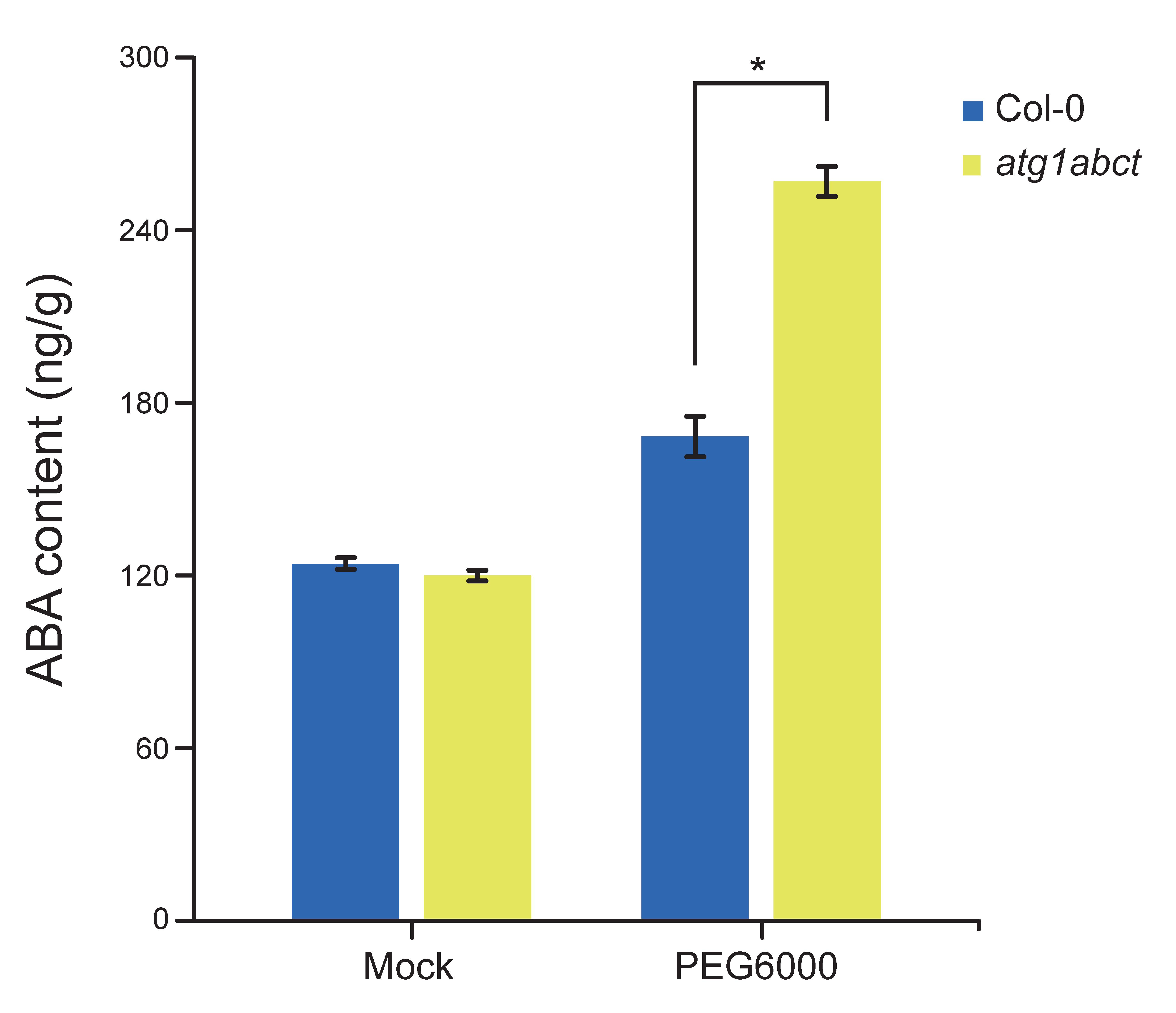


**Figure S4:** Analysis of ABA content in Col-0 and *atg1abct* mutant. 14-day-old Col-0 and *atg1abct* were treated with 20% PEG6000 for 0 h and 4 h, respectively. The error bars represent mean ± SD (n = 3). Significant differences were determined using Student's *t*‐test: **p* < 0.05.
